# The whole mitochondrial genome of *Sarcophaga kanoi* (Diptera: Sarcophagidae)

**DOI:** 10.1080/23802359.2020.1787272

**Published:** 2020-07-02

**Authors:** Yueting She, Chaojun Wang, Weifeng Wang, Qin Zeng, Wei Mao, Xiaoping Gu, Hao Yuan, Xiaoling Pan, Yong Wang

**Affiliations:** a The Key Laboratory of Model animals and Stem Cell Biology in Hunan Province (2019TP1035), School of Medicine, Hunan Normal University, Changsha, P. R. China; b Department of Forensic Science, School of Basic Medical Science, Central South University, Changsha, P. R. China

**Keywords:** Sarcophagidae, *Sarcophaga kanoi*, mitochondrial genome

## Abstract

*Sarcophaga kanoi* Park, 1962, a widely distributed flesh fly in Southeast Asia, is important in forensic entomology. Notably, its mitochondrial genome could provide the unique and accurate molecular information in species identification which facilitate forensic practices in estimation of postmortem interval especially in putrefied cadaver cases. Thus, we sequenced and characterized the whole mitochondrial genome (mitogenome) of *S. kanoi* for the first time, which was collected from Southern China in this study. The 15,319 bp mitogenome contained 13 protein-coding genes (PCGs), 22 transfer RNA genes (tRNA), 2 ribosomal RNA genes (rRNA), and a putative control region. The total nucleotide composition of this circular genome was 39.7% for A, 9.3% for G, 14.2% for C, and 36.8% for T. To better understand the genetic relationship, the phylogenetic analysis was constructed according to the 13 PCGs sequence of *S. kanoi* and other 11 species. The phylogenetic tree showed that the *S. kanoi* was placed in a sub-clade with *S. similis*. Our study updated the new genetic information for dipteran mitogenomes, which could broaden the background knowledge for forensic entomology, molecular genetics and developmental biology. It has the potential application on the species identification using genetic markers.

*Sarcophaga kanoi* Park, 1962 (Roskov et al. 2017), a flesh fly distributed in Asia and Eastern Europe, has been reported presence in northwest, northeast and South-central China. It belongs to the Sarcophagidae family and Diptera order, which is a potential vector of human diseases. Currently, its density is increasing with the elevated levels of organic matter waste produced by human activities and animal breeding, which presents a worldwide health concern (Greenberg 1965). Moreover, its mitochondrial genome makes the essential contribution on the accurate postmortem interval (PMI) estimation in forensic entomology (Harrison 1989, Harvey et al. 2008). However, the complete mitogenome of *S. kanoi* has not been characterized previously. Therefore, we aim to identify the new genetic information. The sample collection of flesh fly was conducted in Hunan province, China. In this study, we firstly reported a whole mitogenome of *S. kanoi* (GenBank ID is MT476487).

The *S. kanoi* sample was collected at Changsha city, Hunan province, P. R. China (28.25°N, 112.55°E) in 9 November 2019. The flies were kept in the laboratory at −80 °C (Changsha, Hunan, China) with a sole code (CSU2020050791). The specimens were identified according to the key morphological characteristics. Subsequently, the genomic DNA was extracted using the QIANamp Micro DNA Kit as manual recommendation. The complete mitogenome was sequenced on an Illumina HiSeq 2500 Platform.

Using the MITObim V1.9 in *de novo* assembling and annotation, the assembled mitogenome was 15,319 bp in size. The heavy strand of the mitogenome molecule consisted of 39.7% adenine (A), 14.2% cytosine (C), 9.3% guanine (G) and 36.8% thymine (T). Totally, the mitogenome contained 13 genes encoding proteins of the respiratory chain, 22 tRNA genes and 2 rRNA genes, and a non-coding AT-rich region. The gene order of the mitogenome of *S. kanoi* was similar to that of other Sarcophagidae tribe whose sequence data are available.

To clarify the genetic relationship of *S. kanoi* with other sarcophagids species, 13 PCGs sequences of *S. kanoi* and other 11 sarcophagids species were chosen to calculate genetic distances and construct phylogenetic tree using the neighbor-joining method via the software MEGA 7.0 (Kumar et al. 2016). The results indicated that the *S. kanoi* was close to *S. similis* (genus *Sarcophaga*) in genetic relationship while *Calliphora vomitoria* and *Chrysomya pinguis* were selected as the root of phylogenetic tree (Figure 1). The classification based on mitochondrial gene sequence is consistent with the results of traditional morphological classification. Our study updated the new genetic information for dipteran mitochondrial genomes, which could broaden the background knowledge for forensic entomology, molecular genetics and developmental biology. It is useful in evaluation of new genetic markers that have the potential application on the species identification.

**Figure 1.**
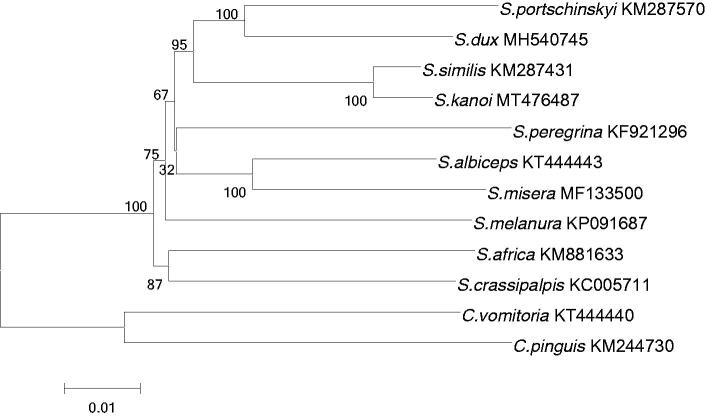
Phylogenetic analyses of 12 sarcophagids species were constructed using NJ method based on 13 PCGs. Morphological species identification and GenBank ID were given in the label. Numbers on branches showed the bootstrap support value. The out-group consists of two species of *Calliphora vomitoria* and *Chrysomya pinguis*.

## Data Availability

The data that support the findings of this study are openly available in GenBank at https://www.ncbi.nlm.nih.gov, reference number MT476487.
